# Long term outcomes of chronic pain patients attending a publicly funded community-based interdisciplinary pain program in the Greater Toronto area: results of a practice-based audit

**DOI:** 10.1186/s41687-022-00452-z

**Published:** 2022-05-07

**Authors:** Angela Mailis, Amol Deshpande, S. Fatima Lakha

**Affiliations:** 1 Pain and Wellness Center, 2301 Major Mackenzie Drive West Unit 101, Vaughan, ON L6A 3Z3 Canada; 2 Department of Medicine, University of Toronto, Toronto, L6A 3Z3 Canada; 3 Toronto Rehabilitation Institute, UHN, Toronto, L6A 3Z3 Canada

**Keywords:** Chronic pain, Interdisciplinary treatment, Outpatient

## Abstract

**Background:**

Chronic pain management multi/interdisciplinary programs attempt to address all elements of the biopsychosocial model. The primary objective of this retrospective study (based on practice-based audit) was to determine the effectiveness of a patient-centered, comprehensive and intense interdisciplinary pain management program in a publicly funded community-based pain clinic in the Greater Toronto Area.

**Method:**

This retrospective longitudinal study was conducted on 218 carefully selected sequential chronic pain patients, with 158 completing a 3–4-month interdisciplinary program between January 2016 and December 2018. Data collected upon exit, at 6 months and 12 months post-discharge included demographic information, pain characteristics, emotional/functional status obtained by validated instruments and global impression of change (GIC). Additionally, social health outcomes (return to work or school) were retrieved through retrospective chart review. Means of pre-and post-program variables were compared to assess changes of each patient’s “journey”.

**Results:**

Physical and mental/ emotional health outcomes at exit, 6 months and 12 months post-discharge, showed initial and sustained, statistically and clinically significant improvement from pre-treatment levels, with GIC (much/very much improved) reported as 77%, 58% and 76%, respectively. Additionally, a substantial positive change in social health outcomes was noted particularly in patients on disability (29%), part time workers gaining full time employment (55%), and students (71%) who improved their level of schooling.

**Conclusion:**

The study showed that careful patient selection in a community-based publicly funded interdisciplinary pain management program can produce significant improvement in pain, physical, mental/emotional health and social function, with sustained long-term outcomes.

## Introduction

Chronic pain is a major societal issue. One in 5 people in Canada suffer from chronic pain [1] with estimated annual cost of $50–60 billion CAD. The prevalence of chronic pain increases steadily with age in adults; children and young teens are not spared, while women, indigenous populations, veterans, drug users etc. are disproportionately affected [1].

While the biomedical reductionist model of treating chronic pain conditions is outdated^2^, the biopsychosocial model of pain and disability (first introduced by Engel 1977) [2] is now widely recognized and postulates that chronic pain and disability are the product of a dynamic interaction of medical, psychological and socioenvironmental factors which generate, perpetuate and worsen each other, reduce quality of life and lead to complex chronic pain syndromes [3].

The biopsychosocial model has led to the development of a multifaceted assessment and treatment paradigm of chronic pain [4]. Gatchel et al. [3] make a clear distinction between *multidisciplinary* versus *interdisciplinary* pain management. While both types of programs use multimodal treatments and a variety of clinicians, the latter is characterized by shared common rehabilitation philosophy, robust ongoing communication between health care professionals, and active patient involvement. Schatman [5], summarized the literature illustrating that interdisciplinary pain programs have a stronger evidence base for efficacy, cost effectiveness, and lack of iatrogenic complications. However, lack of funding is one of the most significant barriers in accessing interdisciplinary pain management programs [3, 6].

Review of the literature demonstrates great variability in inclusion/exclusion criteria for admission to pain management programs. Bosy et al. [7] included patients who suffered from chronic non- malignant pain, had poor response to previous therapies, and were able to attend an outpatient programme. Gilliam et al. [8] included patients with pain in one or more anatomical sites; pain been the predominant focus of the clinical presentation, of sufficient severity to warrant clinical attention. Additionally, they excluded patients with cancer pain, active moderate or severe alcohol or other substance use disorder. Katz et al. [9] included patients with chronic pain who “had no risk for falls and were interested in attending the program”. Knight et al. [10] to the contrary, excluded 44.7% of 200 consecutive patients because of lack of openness to self-management, complex psychopathology, refusal to participate due to family commitments, failing to meet minimum physical criteria/ ability to attend program, low pain interference, language issues, or medical issues.

In Ontario (the largest province of Canada), the Ministry of Health and Long Term Care (MOHLTC) created in 2011 a Chronic Pain Working Group which produced a White (policy) Paper, pointing to a long number of problems in the province and outlined the essential elements needed to support a comprehensive pain system. Details of the process have been published elsewhere [6].

In 2014, MOHLTC provided $18 M CAD in base funding to support interdisciplinary chronic pain management in Ontario across five pediatric hospitals, 13 adult hospitals, and one community chronic pain clinic (the Pain and Wellness Centre or PWC for short, serving so far as the “demonstration project” of MOHLTC in the community). Since MOHLTC has indicated they plan in the future to expand funding outside the academic hospital network to community settings, PWC patient outcomes are of great importance to MOHLTC for future policies.

The PWC is located 45 km north of Toronto in the Greater Toronto Area encompassing 7124 km^2^ with a population of 5.93 M. The PWC offers pain consultations, recommendations and/or medical management to patients referred by community family doctors and specialists, as well as interdisciplinary pain management (IDP) to a select group of chronic pain patients considered appropriate candidates (details are provided in “Methods”). The latter provides medical and pharmacological management as needed, combination of allied health services (psychology, mindfulness, nutritional and dietary counselling, massage therapy including lymphatic drainage, manual and exercise therapy) and access to a community resource facilitator (community navigator). Details about the PWC and the IDP program have been published elsewhere [6].

The PWC pain team elected to offer the program to patients that were considered to have a better chance of responding. High numbers of drop-out patients in certain groups early on led to specific admission criteria explained in the method section.

The present study is the result of an internal evaluation (audit) of our interdisciplinary pain management program in an effort to ascertain its long-term effectiveness and assist MOHLTC in future policy and funding decisions. The primary objective of this study, therefore, was to determine the effectiveness of our community-based interdisciplinary pain management program. The study is unique in the sense that it is (a) pragmatic, evaluating the effectiveness of interventions in real-life routine practice conditions; while (b) funding of the interdisciplinary program is not an obstacle as it is provided by MOHLTC, which allows us to select all the patients who meet our eligibility criteria. Lack of funding is considered one of the most important barriers in provision of interdisciplinary pain management programs, a factor thankfully absent from the present study [3].

## Methods

### Study design

The current study is a retrospective longitudinal pragmatic study of 218 consecutive new patients offered admission to the IDP over a 3-year period (January 2016–December 2018), with the data arising from an internal audit. The study was approved for retrospective analysis by the University of Toronto Research Ethics Board (Protocol Approval #36903). Patients were derived from the general population of patients seen in PWC and met the following criteria published in a previous study [6]: Distance of no more than 50 km (30 Mi) from the clinic (in order to be able to drive or commute frequently, particularly in inclement weather) though exceptions are granted on an individual basis; good command of verbal/written English; no major untreated or poorly treated psychopathology (morbid depression, significant suicidal ideation, disabling anxiety) or life crisis (such as acrimonious divorce, custody battle etc.); high levels of motivation and commitment as judged by the interaction with consulting MD and staff; and availability to attend the clinic 3 times a week for a minimum of 2 h per session for 3–4 months.

### Population, setting and data collection

The study population was derived from the general population of pain patients referred to the PWC for a medical pain consultation. All patients are evaluated by a physician and another team member through an exhaustive history and thorough physical examination; 1–2 patients out of 5 meet established eligibility criteria and are offered admission to our IDP. These patients suffer mostly from myofascial or mechanical pathology, as well as from emotional and cognitive issues secondary to persistent chronic pain, poor coping mechanisms, poor understanding of mind/body interactions etc.

The PWC IDP treatment program is patient centered, interdisciplinary and based on the shared decision-making model [11]. It provides 60–120 h of instructions in a one-to-one format (3 times/week, 2 h/session, over a period of 3–4 months), with the exception of Mindfulness that is offered both in small groups and one-to-one format. The participants are treated by a variety of health professionals (manual/exercise therapists/chiropractors twice/week for the duration of the program; naturopathic doctors 4–6 visits or more; massage therapists once weekly; psychologist once weekly, and Mindfulness facilitator once weekly, while pain physicians are present in the premises at all times and see the patients as needed). The pain team addresses physical, psychological, emotional, nutritional, medical etc. needs, including issues with sleep, weight etc. Medications are modified as needed or tapered/discontinued by the pain physicians Very frequent communication between team members occurs via multiple means, while all notes are uploaded in the Electronic Medical Records system within 24–48 h. Referring providers are kept in the circle of care through copies of medical consultations and medical follow up notes summarizing medical and treatment progress.

Data collection for the study population was electronic, overseen by staff at the time of the intake interview and included (a) demographics such as age, sex, marital status, work status, country of birth and education; (b) individual scores of validated questionnaires at baseline, exit and 6 and 12 months post exit (physical and mental/emotional health outcomes); and (c) social health outcomes (i.e. employment, schooling etc.), the latter through retrospective chart review, documenting social status at the time of entry to the program vs social status at 6 and/or 12 months. Multiple reminders were sent to patients regarding completion of the questionnaires at 6 and 12 months after program exit. Follow up medical visits continued after exit from the IDP (which allowed us to collect social health outcomes several months after termination of the pain management program).

### Outcome measures

Based on the literature on chronic pain management outcomes [9], a number of questionnaires were selected to capture physical and emotional/ mental outcomes reflecting the goals of the program to reduce pain disability and emotional distress (depression, catastrophizing, anxiety, fear of movement).

The validated questionnaires we used included the following: pain catastrophizing scale (PCS) [12]; pain self-efficacy scale (PSEQ) [13]; Tampa scale for kinesiophobia (TSK) [14]; general anxiety disorder questionnaire (GAD) [15]; Centre for Epidemiological Studies—Depression (CESD) [16]; and brief pain inventory (BPI) [17]. The latter provided data on pain intensity (based on the pain average score on a numerical rating scale—NRS); pain interference (arithmetic mean of seven interference items); and Total Pain Severity score (consisting of the sum of NRS worse, least, current, and average pain score). The same battery of validated questionnaires was completed at exit, 6, and 12 months after program termination, together with addition of the Global Impression for Change. The latter assesses patients' own impressions of change, using a global scale from “much better” through “no change” to “much worse”. Since patients themselves make a subjective assessment of the significance of the change in their lives as a result of treatment, this scale is frequently used as the external criterion or “gold standard” of clinically important change [18, 19].

Furthermore, in this study, we extended our efforts to extract social health data relating to patients’ work or school status, which could not be captured by the questionnaires we cited above. Such information could only be obtained through retrospective chart review as the vast majority of patients continued visiting the physicians in our centre. The impact of pain in the workplace is an important issue to be considered in chronic pain patients. Studies carried out in different countries have shown that patients who are affected by pain have high levels of work absenteeism. Not only must they often change their occupational duties or post, but they may even end up losing their job as a result of their pain symptoms [20]. Patients’ social health status in this study was categorised in 7 subgroups: employed full time (FT); employed part time (PT); students; unable to work due to disability; homemakers; retirees; and miscellaneous (volunteer, unemployed looking for a job, maternity leave etc.).

The mean BPI pain intensity (rated in a 0–10 numerical rating scale) and pain interference scores were divided into three categories. A 10–20% decrease in pain intensity and pain interference over the 3 months of the program was considered as a *minimally important improvement*; a decrease ≥ 30% as a *moderately important improvement*; and a decrease ≥ 50% as a *substantial improvement* according to the main IMMPACT outcome recommendations [21].

### Data analysis

All data were analyzed using SPSS (Statistical Package for the Social Sciences v.16.0, SPSS Inc., Chicago, IL, USA). Descriptive statistics were used for demographics, pain, diagnostic groups, and opioid data. The descriptive analysis consisted of means and proportions according to the nature of the variables. For categorical data, proportion and size of each category for all demographic characteristics (such as sex, marital status, education, work status and country of birth), and body map areas, were calculated. Means and standard deviation were computed as well for pain characteristics (such as pain rating, pain interference score, pain duration), opioid dosages and validated questionnaire outcome measures. For statistical comparison of categorical and continuous variables, Pearson χ^2^ test or Fisher’s exact tests and t-tests, were used. For the ratio analysis χ^2^ goodness-of-fit test was used. At 95% confidence interval, the two-sided *p* value of < 0.05 was used to define minimal statistical significance. When the denominator is different due to missing data, the exact number will be indicated in brackets. Means (± SD) of pre-and post-program variables were compared to assess effectiveness of program progress. The percent change in pain intensity and functional and emotional scores was calculated as the difference of mean between baseline, 6 and 12 months.

## Results

A total of 218 patients were enrolled in the IDP and 169 completed the program (completers). Of note, 11 out of 169 completers, did not provide exit questionnaires for various reasons and only their demographic and entry data were used for parts of the analysis. Therefore, exit data analysis was done on n = 158 datasets. Unfortunately, a number of patients could not be located/ contacted despite email, text and phone attempts, or did not fill the forms even when they received verbal, telephone and email reminders, so returned questionnaires for 6 and 12 months were 66 and 46, respectively.

### General demographics and pain characteristics

We noted the following for the entire cohort (N = 218): female predominance (F/M, 2/1); mean age 45 +/− 16; pain duration 8 ± 8 yrs (range 1–35 years); 12% were > 65 years of age; 62% were Canadian born (CB); 13% had chronic pain due to disease (diabetic neuropathy, degenerative disc disease, etc.); and 68% of the patients had sustained some form of compensable trauma with legal involvement (car accident, work, slip and fall). Details are presented in Table 1.

**Table 1:** Demographics of all groups

Demographic variables	Total population N = 218	Completers N = 158	Non-completers N = 49
*Gender*			
(a) Male	70(32%)	50(32%)	17(35%)
(b) Female	148(68%)	108(68%)	32(65%)
*Male: Female*	1:2.1	1:2.16	1:1.8
*Age (mean* ± *std)*	45 ± 16	46 ± 16	43 ± 14
(a) < 65	192(88%)	137(87%)	46(94%)
(b) 65 +	26(12%)	21(13%)	3(6%)
*Place of birth*	N = 217	N = 157	N = 49
(a) Canadian born	134(62%)	105(67%)	25(51%)
(b) Foreign born	83(38%)	52(33%)	24(49%)
*Marital status*	N = 218	N = 158	N = 49
Single, divorced, separated, widow	95 (44%)	64 (41%)	26 (53%)
Married, common law	123 (56%)	94 (60%)	23 (47%)
*Level of education*			
High school or less	81 (37%)	60 (38%)	18 (37%)
College, university, postgraduate	120 (55%)	85 (54%)	28 (57%)
*Annual household income*	N = 214	N = 155	N = 48
Less than $19,999	32(15%)	17(11%)	10(20%)
$20,000 to $49,999	58(27%)	41(26%)	14(29%)
$50,000 to $99,999	77(35%)	61(39%)	13(27%)
$100,000 to $149,999	29(13%)	23(15%)	6(12%)
More than $150,000	18(8%)	13(8%)	5(10%)
*Use of cannabis during the past year* +			
Yes	65(30%)	49(31%)	13(27%)
No	153(70%)	109(69%)	36(73%)
*Are you currently using*	N = 65	N = 49	N = 13
Cannabis Prescribed	27(12%)	21(13%)	5(10%)
Non-prescribed	29(13%)	26(17%)	2(4%)
Both	9(4%)	2(1%)	6(12%)
Pain characteristics	N = 177	N = 133	N = 35
*Pain cause*			
Disease	23 (13%)	18(14%)	3(9%)
*Injuries with medicolegal implications*			
MVA	68(38%)	52(39%)	12(34%)
Work accident	26(15%)	16(12%)	8(23%)
Slip and Fall	26(15%)	19(14%)	5(14%)
Other incl. surgery	34 (19%)	28(21%)	7(20%)
*Pain body map area*			
Lower back/buttocks/hips	143(66%)	110(70%)	28(57%)
*Pain rating (BPI scale)*			
Pain at its WORST in the last week (mean ± std)	8 ± 1	8 ± 1	8 ± 1
Pain at its LEAST in the last week (mean ± std)	4 ± 2	4 ± 2	5 ± 2
Pain you have RIGHT now (mean ± std)	6 ± 2	6 ± 2	6 ± 2
AVERAGE pain in the last week (mean ± std)	6 ± 2	6 ± 2	7 ± 2

+ Data was collected prior to legalization of cannabis in Canada in October 2018

### Characteristics of non-completers

Forty four patients of the 218 enrolled patients, started but failed to complete the program (non-completers) for the following reasons: Multiple No Shows or Late Cancellations (n = 12); Limited availability due to work or other life commitments (n = 8); Medical reasons (n = 6); Interruption due to life crisis that occurred after entry to the program (n = 5); Dismissed by program due to non-compliance (n = 6); Patients dropped out on their own volition (n = 7). Another 5 patients who were enrolled and completed the entry battery, never showed up to start the program (n = 5). Altogether, the non-completer group consisted of 49 patients. With the exception of those patients who interrupted the program due to medical reasons (n = 6), the remaining 43 patients provide a measure of “non-compliance” for reasons cited above. The patients (n = 169) who entered the program and completed it fully were obviously the “compliant and adherent” group.

No demographic differences were found between completers versus non-completers in any of the domains other than country of birth where we documented a statistically significant difference (*p* < 0.05) between Canadian Born (CB) versus Foreign Born (FB). The CB/FB ratio was 2/1 for completers (similar to that reported by Canadian Census for GTA), and 1/1 for non-completers of CB/FB due to significant increase of FB in the latter subgroup (Table 1).

### Physical and mental/emotional health outcomes

No difference was found between baseline and entry responses of completers vs non-completers and females versus males in the scores of PCS; PSEQ; TSK; GAD; CESD; and BPI pain intensity (arithmetic mean of the four severity items: current, average, worst and best) and pain interference (arithmetic mean of the seven interference items) (Table 2).

**Table 2:** Mean (± SD) scores for all patients, completers and non-completers on outcome measures at ENTRY

	PCS	PSEQ	TSK	GAD	CESD	BPI pain severity score (max. 40)	BPI interference score (max. 70)
All Patients N = 218	28 ± 12	25 ± 14	41 ± 9	11 ± 6	24 ± 14	24 ± 6	45 ± 14
Completers N = 158	28 ± 13	25 ± 14	41 ± 8	10 ± 6	24 ± 13	24 ± 6	45 ± 14
Non-completers N = 49	30 ± 11	25 ± 14	40 ± 9	12 ± 6	24 ± 13	26 ± 7	45 ± 14

In terms of physical and mental/emotional health outcomes, improvement (with statistical significance at level 0.001) was noted in completers across all validated scales at exit. Percent change (indicating improvement) between entry and exit scores of all completers was as follows: Pain catastrophizing 46%; Pain self-efficacy 52%; anxiety 40%; depression 50%; kinesiophobia 15%; BPI pain severity 29%; and BPI pain interference score 40% (Tables 3, 4). Table 3 report outcome scores between entry and 6 and 12 month follow up.

**Table 3:** Mean (± SD) score and t-test of completers on outcome measures between entry to exit, 6 and 12-month

Variables Mean ± std	Entry N = 158	Exit N = 158 t(df)	6 Month N = 66 t(df)	12 Month N = 46 t(df)
Pain catastrophizing scale (PCS, 0–52)	28 ± 13	15 ± 12* 13.6(157)	15 ± 13* − 6.8(222)	10 ± 9* − 8.7(202)
Pain self-efficacy questionnaire (PSEQ, 0–60)	25 ± 14	38 ± 15* 10.8(157)	38 ± 8* 7.1(222)	44 ± 14* 8.1(202)
Tampa scale of kinesiophobia (TSK, 17–68)	41 ± 8	35 ± 9* 8.3(157)	36 ± 8* − 4.2(222)	35 ± 7* − 4.6(202)
Generalized anxiety disorder (GAD, 0–21)	10 ± 6	6 ± 5* 10.1(157)	7 ± 6* − 3.4(222)	5 ± 5* − 5.2(202)
Center for epidemiological studies—depressed mood scale (CESD, 0–60)	24 ± 13	12 ± 9* 16.8(157)	16 ± 14* − 4.1(222)	13 ± 9* − 5.3(202)
BPI pain severity score	24 ± 6	17 ± 9* 9.8(157)	16 ± 9* − 7.7(222)	15 ± 9* − 7.9(202)
BPI interference score (0–70)	45 ± 14	27 ± 18* 12.6(157)	24 ± 20* − 8.9(222)	21 ± 16* − 10.0(202)

Statistical significance among group is marked by *. * *p* < 0.0001 level (2-tailed)

**Table 4:** Percent of change (improvement) of completers from baseline/entry to exit, 6 and 12 month

Variables	Exit (%)	6 Month (%)	12 Month
Pain catastrophizing scale (PCS)	46	46	64%
Pain self-efficacy questionnaire (PSEQ)	52	52	76%
Tampa scale of kinesiophobia (TSK)	15	12	15%
Generalized anxiety disorder (GAD)	40	30	50%
Center for epidemiological studies-depressed mood scale (CESD)	50	33	46%
BPI pain severity Score	29	33	37.5
BPI interference score	40	47	53%
Global impression of change (GIC)	77	58	76%

Table 4 summarizes the percent change from entry to exit, 6 and 12 months follow up and includes global impression of change—GIC, reflecting the subjective evaluation of the participants as to their rate of improvement. Aggregate data for physical and mental/emotional health outcomes demonstrated slight reduction of benefit at the 6-month mark, but complete recovery of values at 12 months, i.e., improvement scores reaching the level of those obtained at exit. Based on GIC scores, the proportion of completers who stated they were “much/ very much improved” was 77%, 58% and 76% at exit, 6 months and 12 months post exit, respectively.

Improvement ≥ 50% was experienced at exit by 30% of patients on BPI pain severity score and 41% of the patients on BPI interference score, while worsening of pain score and pain interference was noted in 14% and 16%, respectively (Table 5).

**Table 5:** Percentage of Change (improvement) of Completers at ≥ 50%, ≥ 30%, < 30% or worsening at exit

Variables N = 158	Percentage of Change (Columns # 1–4)			
	1	2	3	4
	≥ 50 improvement	≥ 30 improvement	< 30% improvement	Worse
BPI pain severity score N	48(30%)	78(49%)	80(51%)	22(14%)
BPI pain interference score	65(41%)	98(62%)	60(38%)	26(16%)

Please note that column 1 is subset of column 2 and column 4 subset of column 3

### Social health outcomes

We were able to retrieve social health data for 150/158 completers through collateral information from follow up medical visits, despite decreased numbers of returned patient questionnaires at 6 and 12 months. Detailed analysis of social health status at 6 or 12 months after program termination, demonstrated that after program exit, 26% of the cohort collectively improved their social health status (e.g., obtaining employment, increasing work from part time to full time, returning to full time school or progressing to university etc.). Significant social status improvement was noted in 29% of the patients classified upon entry as disabled, 55% of the part time workers, 71% of students and 75% in the small group of those under the miscellaneous group. Naturally, the social status of homemakers and retirees did not change (even if most experience change in quality of life), while 8% of those who were working full time jobs upon entry to the program got (self-reported) better employment. The results are detailed in Table 6.

**Table 6:** Social health outcomes

Social status at entry	N	Status change at 6/12 months		% improvement/class
FT work	48	Better job	4	8%
		Missing data	4	
		Different*	3	
		Worse	1	
		Unchanged	36	
PT work	18	FT work	10	55%
		Missing data	2	
		Different**	22	
		Worse	1	
		Unchanged	3	
Disabled	48	FT work	7	29%
		PT work	6	
		Back to school	1	
		Unchanged	34	
Student	14	Improved (from PT to FT, advancing to university etc.)	10	71%
		Missing data	2	
		Unchanged	2	
Miscellaneous***	4	Improved (return to work, school etc.)	3	75%***
		Missing data	1	
Homemakers	8	Unchanged	8	–
Retirees	18	Unchanged	18	–
Total	158		158	

*The designation indicates status change due to new event (laid off, work searching, new car accident)

**The designation indicates change in status due to new event (surgery, childbirth)

***Miscellaneous (volunteer, work searching, maternity leave etc.)

## Discussion

This study presents long-term data (up to 12 months post-graduation) from a series of well selected chronic pain patients admitted to a Canadian community-based, comprehensive, one-to-one, interdisciplinary pain management program funded by MOHLTC. The study population consisted of middle age, well-educated chronic pain patients (with female predominance), with a high proportion of employment (full and part time, 42%, Table 6). Physical and mental/ emotional health outcomes showed statistically and clinically significant initial and sustained improvement with GIC (much/very much improved) at discharge, and 6-months and 12 months from pre-treatment levels, of 77%, 58% and 76%, respectively. Additionally, a substantial positive change in social outcomes was noted particularly in patients who were upon entry to the program on disability due to chronic pain conditions, part time workers gaining full time employment, and students who improved their level of schooling (e.g. from part time to full time, high school to university etc.).

The high proportion of chronic pain patients still at work upon admission to our program is due to early referral by primary care physicians triggered by deterioration at the workplace with decreased productivity and increased absenteeism. We believe that our early intervention maintained the ability of these patients to remain at work or increase their work output.

Our attrition rate (22.4%) is consistent with that reported in the literature (17–57%) [7, 10, 22]. There were no differences in demographics and entry scores on physical and mental/ emotional health between those who completed the program and non-completers other than country of birth for foreign born non-completers (potentially reflecting cultural, employment, household obligations, family structure and psychosocial support differences).

The poor completion rate of validated batteries at 6 and 12 months is a well-known phenomenon and has been reported in pragmatic practice-based clinical studies [7, 8, 23]. Despite our repeat attempts to have the patients complete the follow up questionnaires via text, phone call and email reminders, we had a high number of participants who failed to complete the follow up questionnaires (while the vast majority attended follow up medical visits to our centre, hence we were able to retrieve social health outcomes). The low response rate to our validated batteries may possibly skew our long-term results; however, collateral information from the charts regarding social outcomes in 150/158 completers, strongly suggests that long term benefits from the program are maintained by the majority of patients.

The slight drop in the subjective GIC “much/ very much improved” scores at the 6-month period after program termination and subsequent improvement to exit levels at 12 months, has been observed in other programs. While the reduced number of returned questionnaires at 6 and 12 months may have skewed the results, we did review our raw data and realized that depression and anxiety had increased at 6 months (accounting for the GIC drop), consistent with the hypothesis of an “adjustment period” [22] until skills are consolidated and life goals get reset (at 12 months) with concomitant increase of subjective GIC “much/ very much improved” scores, equal to those observed at exit.

Our IDP program meets all the criteria of a well-functioning interdisciplinary pain program (as per the American Pain Society White Paper: Interdisciplinary Pain Management, 2008), summarized by Stanos [22] as follows: Shared philosophy, mission, and objectives; Patient and family centered; Providers/therapists work together for common, agreed upon goals; Approach is integrated and interdependent; Mutual respect and open communication exists between providers, often in team meeting format; Communication is frequent, effective, direct, clear, and reciprocal among team members as well as with primary care providers and referral sources; Quality improvement efforts are ongoing from all team members; Approach to clinical care, education, quality improvement, and research is collaborative; and multimodal treatments are employed.

Our thematic analysis (IASP poster 2020) [24] has shown that the main reasons that patients recommend to others our interdisciplinary pain management program are: (1) support and validation provided by the interdisciplinary team; (2) the acquisition of pain management tools, namely strategies and techniques on a variety of domains (physical, emotional, cognitive, nutritional etc.); and (3) personalized one-to-one management plan to improve overall quality of life alongside pain control.

Our eligibility criteria have evolved over time after we observed during the first months of the program high drop-out rates in patients with severe untreated or poorly treated psychopathology or presence of life crisis (though the latter can still happen in the midst of the program as our data show). Besides absence of psychopathology, patient motivation is a primary driver for admission, judged not through formal questionnaires, but during a detailed 2–2.5 h team assessment, and if required, review by a psychologist before the patient is accepted to the program. Given the high number of foreign-born patients in the non-completer group, we now pay more attention to family obligations and support that may vary based on cultural background. Our eligibility criteria ultimately are very similar to those of Knight et al. [10].

Existing literature shows considerable variability in interdisciplinary pain management programs in regard to eligibility criteria, program duration, treatment service provision, mode of treatment administration (primarily group and rarely one-to-one, or combination), funding of the services, numbers of treatment hours etc. [7, 9, 22, 23, 25]. However, all programs irrespective of operational model show improvement in physical, mental and/or social health, though the degree and duration of benefits varies.

Deckert et al. [26] conducted a systematic literature review and summarized all reported outcome domains in 70 studies meeting selection criteria and assessing the effects of multimodal pain treatments (MPT) for chronic pain. There were twelve single outcome domains identified: three for physical health outcomes, e.g. pain, disability and physical function; six for mental/emotional health outcomes, e.g. fear, depressive symptoms, psychological distress, coping, self-efficacy and catastrophizing; and three for social health outcomes, e.g. work ability, sick leave and actual work status. On the other hand, IMMPACT consensus (Initiative on Methods, Measurement, and Pain Assessment in Clinical Trials) includes the following Core Outcome Domains: (1) pain, (2) physical functioning, (3) emotional functioning, (4) participant ratings of improvement and satisfaction with treatment, (5) symptoms and adverse events and (6) participation disposition [27]. However, IMMPACT does not include measurements of the social dimension of the bio-psycho-social approach of MPT (e.g. return to work or work ability), and this partially explains why there is little overlap between IMMPACT domains and outcome domains used in MPT studies [26].

Clearly our program provides long term outcome data on all three domains (physical, mental/emotional and social health).

## Study limitations

Limitations of the study given the specific structure of our program limiting generalizability are:

- Our patient selection criteria result in selection bias, as we enrol the best patients that can benefit from our program.
- Since MOHLTC funding is limited to a single community-based pain management program (ours), our results are not generalizable to other pain management facilities that depend exclusively on third-party funding as such programs are costly and third parties must approve funding. Therefore, many pain patients who can benefit from these treatments can not access them.
- Our program offers nearly exclusively one-to-one in-person treatment. In contrast, the vast majority of programs reported in the literature offer mostly or exclusively group sessions in-person, and some partial or exclusive virtual group sessions. In this context, it would be difficult to exactly duplicate our treatment model.
- Treatment services and hours in our program vary substantially from 60 h (for 3 different forms of treatment) to 120 h (for all treatment services and occasionally additional services during transitional periods). The program is totally customized for each participant and tailored to specific patient needs and goals. Such customization is not possible in many interdisciplinary pain management programs.
- Our patients have access to the physicians on site for their medical needs during the program, while they have follow-up visits (regular or as needed) after they exit the program. This provides continuity of care and contrasts many other interdisciplinary programs where follow ups in general are limited or terminate shortly after the program ends.
- Furthermore, the decreased numbers of returned questionnaires at 6 and 12 month may have limited the validity of the follow-up analysis (though long term social health data for almost all participants support long term improvement). This phenomenon plagues pragmatic practice-based studies, and we attempt harder now to address it by intensifying our telephone, email and text reminders.

## Conclusions

Careful patient selection in a publicly funded, patient-centered, comprehensive and intense interdisciplinary pain management program produces significant improvement in pain, physical, mental/emotional health and social function, with sustained long-term outcomes (and significant functional gains transferable to the workplace or school setting).

While most interdisciplinary pain management programs are conducted in large tertiary care academic settings, our study shows that such programs can be successfully delivered in a community setting, while secure funding ensures treatment of all eligible patients and not just those “who can afford” the program.

Our study model and results have wide implications for MOHLTC policymakers in their effort to reduce personal suffering and the economic burden of chronic pain by expanding public funding to community-based pain clinics in Ontario.

## Data Availability

Data is not available due to patients/participants’ privacy and confidentiality that could be compromised.
